# Experiences in treatment of multiple sclerosis with natalizumab from a real-life cohort over 15 years

**DOI:** 10.1038/s41598-021-02665-6

**Published:** 2021-12-02

**Authors:** Michael Auer, Anne Zinganell, Harald Hegen, Gabriel Bsteh, Franziska Di Pauli, Klaus Berek, Elena Fava, Sebastian Wurth, Thomas Berger, Florian Deisenhammer

**Affiliations:** 1 Department of Neurology, Medical University of Innsbruck, Anichstraße 35, 6020 Innsbruck, Austria; 2 Department of Neurology, Medical University of Vienna, Währinger Gürtel 18-20, 1090 Wien, Austria; 3 Department of Neurology, Medical University of Graz, Auenbruggerplatz 22, 8036 Graz, Austria

**Keywords:** Immunology, Neuroscience, Medical research, Neurology

## Abstract

Natalizumab (NTZ) has been used for treatment of highly active relapsing–remitting multiple sclerosis (MS). When stopping NTZ the risk of severe rebound phenomenon has to be considered. We aimed to investigate the use of NTZ in clinical routine and focused on identification of potential risk factors for disease reactivation after treatment discontinuation. At the Medical University of Innsbruck, Austria, we identified all MS patients who were treated with NTZ and performed a retrospective analysis on therapeutic decision making, disease course before, during and after treatment with NTZ and on risk factors for disease reactivation after NTZ discontinuation. 235 NTZ treated MS patients were included, of whom 105 had discontinued treatment. At NTZ start disease duration was 5.09 (IQR 2.09–10.57) years, average number of total relapses was 4 (IQR 3–6) and median EDSS 2.0 (range 0–6.5), whereby these values significantly decreased over time. Reduction of annualized relapse rate (ARR) on treatment was 93% and EDSS remained stable in 64%. In multivariate regression models only conversion to secondary progressive MS (SPMS) on treatment was significantly associated with lower risk of disease reactivation after NTZ, while ARR before treatment was associated with earlier disease reactivation. We could confirm the high therapeutic efficacy of NTZ which trends to be used earlier in the disease course nowadays. Discontinuation of NTZ seems safe only in patients who convert to SPMS during treatment, while higher ARR before NTZ increases the risk of disease reactivation after treatment discontinuation.

## Introduction

Natalizumab (NTZ) is a humanized monoclonal antibody used for treatment of highly active relapsing remitting multiple sclerosis (MS). The mechanism of action is based on blocking α4 integrin (very late antigen 4) on the surface of lymphocytes so that the interaction with the vascular cell adhesion molecule 1 and the consecutive migration of autoreactive lymphocytes through the blood–brain barrier is reduced^1^. The safety and efficacy of NTZ was approved in two large randomized, controlled studies (AFFIRM, SENTINEL) showing a reduction of annualized relapse rate (ARR) of approximately 70%^2,3^. NTZ was finally approved by the US Food and Drug Administration as well as in the European Union in 2006^1^. Since that time, NTZ has been increasingly used in patients with a highly active disease course with at least two relapses within  one year and according magnetic resonance imaging (MRI) findings, or mostly in patients where other disease-modifying treatments (DMT) like interferon-beta and glatirameracetate did not demonstrate sufficient therapeutic effectiveness^4^. With stratification tests for anti-JC virus (JCV) antibodies^5,6^ and the discovery of the dependence of risk of progressive multifocal leukoencephalopathy on treatment duration^7,8^ and JCV antibody index^9^, new guidelines for monitoring NTZ-treated MS patients were introduced^10,11^. Since new, highly effective therapeutic options were approved in recent years and awareness has been raised regarding the risk of disease reactivation after NTZ discontinuation^12,13^, safe strategies for switching from NTZ to other DMTs^14^, such as B cell-depleting therapies^15^, have been sought. Extended interval dosing (EID)^16^, shorter intervals of treatment interruption after NTZ, short-term MRI monitoring during DMT switch and preference for certain DMTs have been discussed in order to avoid a rebound of the disease^17,18^. Furthermore, risk of disease reactivation has to be discussed in the context of female and male MS patients of childbearing age who wish for a child, because use of NTZ for this group is still a subject of debate^19–22^.

While NTZ is a well-studied drug with reliable data from clinical trials, analysis of a real-life cohort could add important insights about the use of NTZ in clinical routine and how the disease develops before, during and after NTZ treatment and could identify risk factors for disease reactivation after stopping NTZ.

## Results

**Figure 1:**
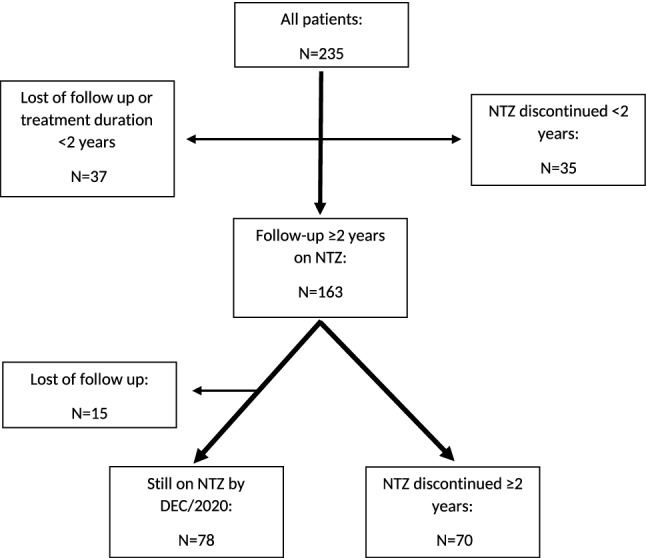
Cohort of patients treated with NTZ, flow chart. Flow chart of all MS patients at the University hospital of Innsbruck, Austria, treated with NTZ from 2006 to 2020. All these selected patients (N = 235) were eligible for the pre-treatment analysis. For the on-treatment analysis patients with a minimum treatment duration of 2 years were considered (N = 163) by excluding those patients who were lost of follow-up or discontinued NTZ within less than 2 years or were still on NTZ by DEC/2020 but with a treatment duration less than 2 years. The discontinuation analysis was performed on all patients who discontinued NTZ before or after a treatment duration of 2 years (N = 105).

At the Medical University of Innsbruck (MUI), Austria, from 2006 to December 2020, a total of 242 patients were treated with NTZ, all of whom had a relapsing–remitting disease course at treatment start. Seven patients had to be primarily excluded because sufficient data for analysis were not available. This resulted in a study cohort of 235 patients. Figure 1 shows the number of patients who stayed on treatment, discontinued NTZ or were lost to follow-up. Of these 235 patients, 181 (77.0%) were female and 54 (23.0%) male.

### Pretreatment analysis

A total of 235 patients were included for pretreatment analysis. The median age at NTZ start was 32.8 (interquartile range [IQR] 25.4–39.3), and the median disease duration before NTZ start was 5.09 (IQR 2.09–10.57) years. Since the stratification test for anti-JCV antibodies was introduced in 2010, JCV status at treatment start was available for 196 patients excluding those 39 patients who started NTZ before the JCV testing era. Of these, 90 (45.9%) were positive for anti-JCV antibodies at treatment start. Detailed data of treatment history before NTZ were available for 232 patients and are displayed in Table 1.

**Table 1:** Disease modifying treatment (DMT) before NTZ.

	N (%)
**(A) Number of DMTs before NTZ**	
0	19 (8.2)
1	135 (58.2)
2	56 (24.1)
3	16 (6.9)
4	5 (2.2)
5	1 (0.4)
**(B) DMTs before NTZ**	
IFN-beta	192 (56.6)
Glatirameracetate	59 (17.4)
IVIG	34 (10.0)
Dimethylfumarate	14 (4.1)
Fingolimod	7 (2.1)
Teriflunomide	4 (1.2)
Cyclophosphamide	3 (0.9)
Mitoxantrone	2 (0.6)
Azathioprine	2 (0.6)
Alemtuzumab	1 (0.3)
Rituximab	1 (0.3)
Ciclosporine	1 (0.3)

A: Number of different DMTs before treatment start with NTZ in a total of 232 patients.

B: Type of DMT before treatment start with NTZ. All DMTs per patient administered before NTZ were considered, resulting in 339 DMTs in 213 patients with pre-treatment.

*DMT* disease modifying treatment, *IFN* interferon, *IVIG* intravenous immunoglobulins.

**Figure 2:**
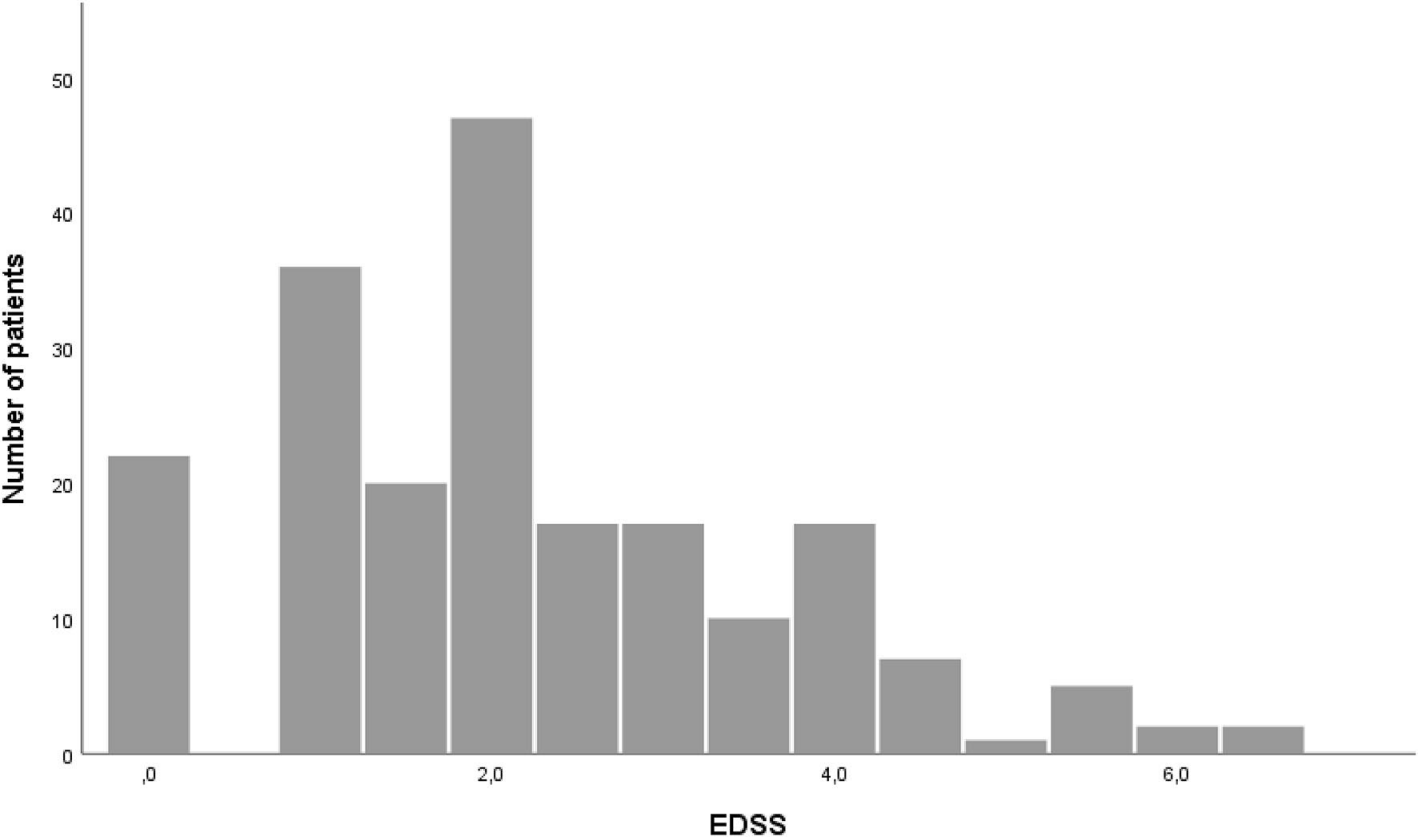
EDSS score at treatment start with NTZ.

**Figure 3:**
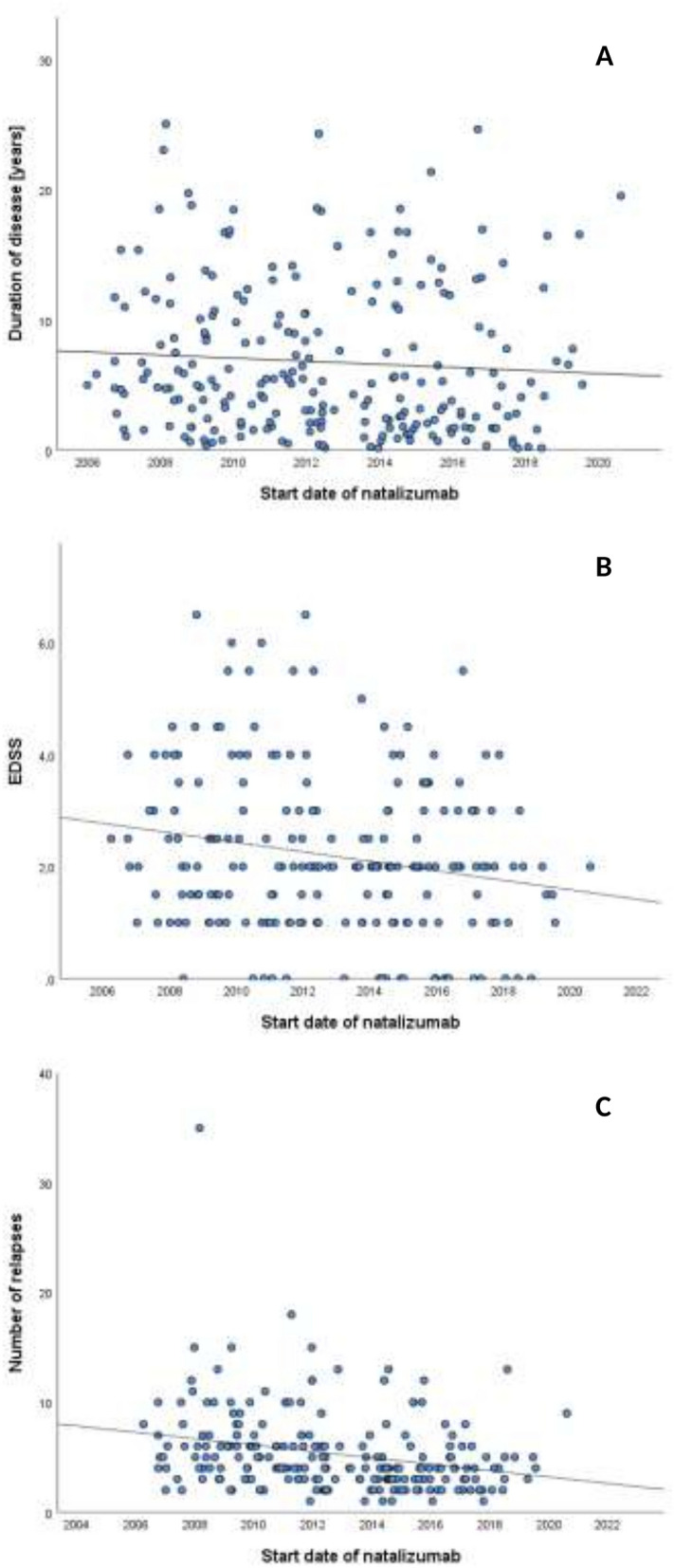
Scatter blots representing regression models of disease duration, EDSS score and number of relapses before start of NTZ over time. Linear regression models of disease duration (**A**), EDSS score (**B**) and number of previous relapses from disease onset (**C**) at time of NTZ treatment start from 2006 to 2020. EDSS score at NTZ treatment start and number of relapses before NTZ significantly decreased over time reflecting change of clinical practice. Significance (p) and Pearson correlation coefficient (r) of regression models: (**A**) p = 0.139, r = − 0.71. (**B**) p = 0.002, r = −0.198. (**C**) p < 0.001, r = − 0.263.

The median Expanded Disability Status Scale score (EDSS) at NTZ start was 2.0 with a range between 0 and 6.5; details are shown in Fig. 2. Before NTZ start, patients experienced a median of four relapses (IQR 3–6) from disease onset, and the median ARR was 1.01 (IQR 0.60–1.78). Change of disease duration, EDSS score at treatment start and relapses before NTZ from the introduction of NTZ in 2006 to the present are shown in Fig. 3 and revealed a significant decrease of EDSS (p = 0.002, r = − 0.198) and relapses (p < 0.001, r = − 0.263) before NTZ start over time.

### On-treatment analysis

**Figure 4:**
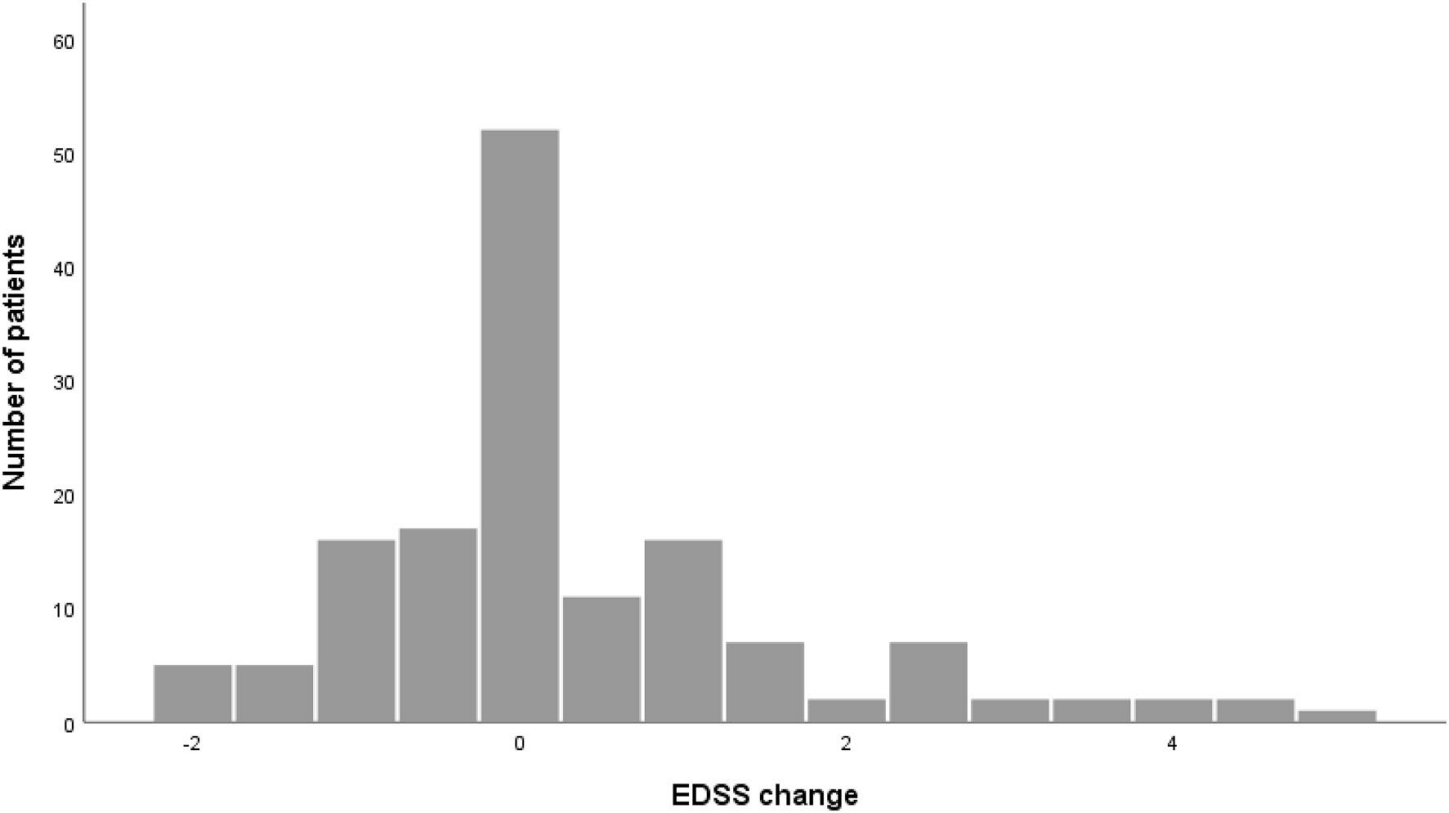
EDSS change on treatment with NTZ. Change of EDSS from time of NTZ treatment start to last visit on treatment for patients with treatment duration ≥ 2 years.

A total of 163 patients were eligible for on-treatment analysis. The mean treatment duration was 6.27 ± 3.27 years for patients who were still on treatment and had follow-ups until December 2020 and 3.35 (IQR 1.50–6.76) years for patients who discontinued NTZ. In the cohort of patients with at least 2 years of follow-up, the reduction of ARR was 93% (p < 0.001, Z = − 10.82, r = − 0.83), and the median ARR decreased from 1.03 (IQR 0.61–1.73) to 0 (IQR 0–0.13). Figure 4 shows change of EDSS score during the treatment period for patients who remained on NTZ for at least 2 years. EDSS data from 147 patients were available. Ninety-four (63.9%) of these patients showed a stable or confirmed improvement of EDSS score during the whole treatment period, whereas 42 (28.6%) experienced a confirmed EDSS progression of at least one point during treatment. The median annualized EDSS progression was 0 (IQR − 0.07 to 0.14), and there was no difference between patients who continued and discontinued NTZ in terms of this analysis. A follow-up of anti-JCV antibodies during treatment was available for 187 patients. Fourteen (7.5%) of these patients converted from initially negative to positive anti-JCV antibody status. In the whole NTZ cohort, three cases of PML were observed. Of all NTZ-treated patients, 40 received EID at any time, which means extension of intervals between NTZ infusions to five to eight weeks. The median EID period was 0.82 (IQR 0.42–3.63) years, and the median ARR on EID was 0 (IQR 0–0.15). Of the 40 EID patients, only four (10%) experienced a relapse during EID, and 22 discontinued NTZ thereafter.

### Discontinuation analysis

**Figure 5:**
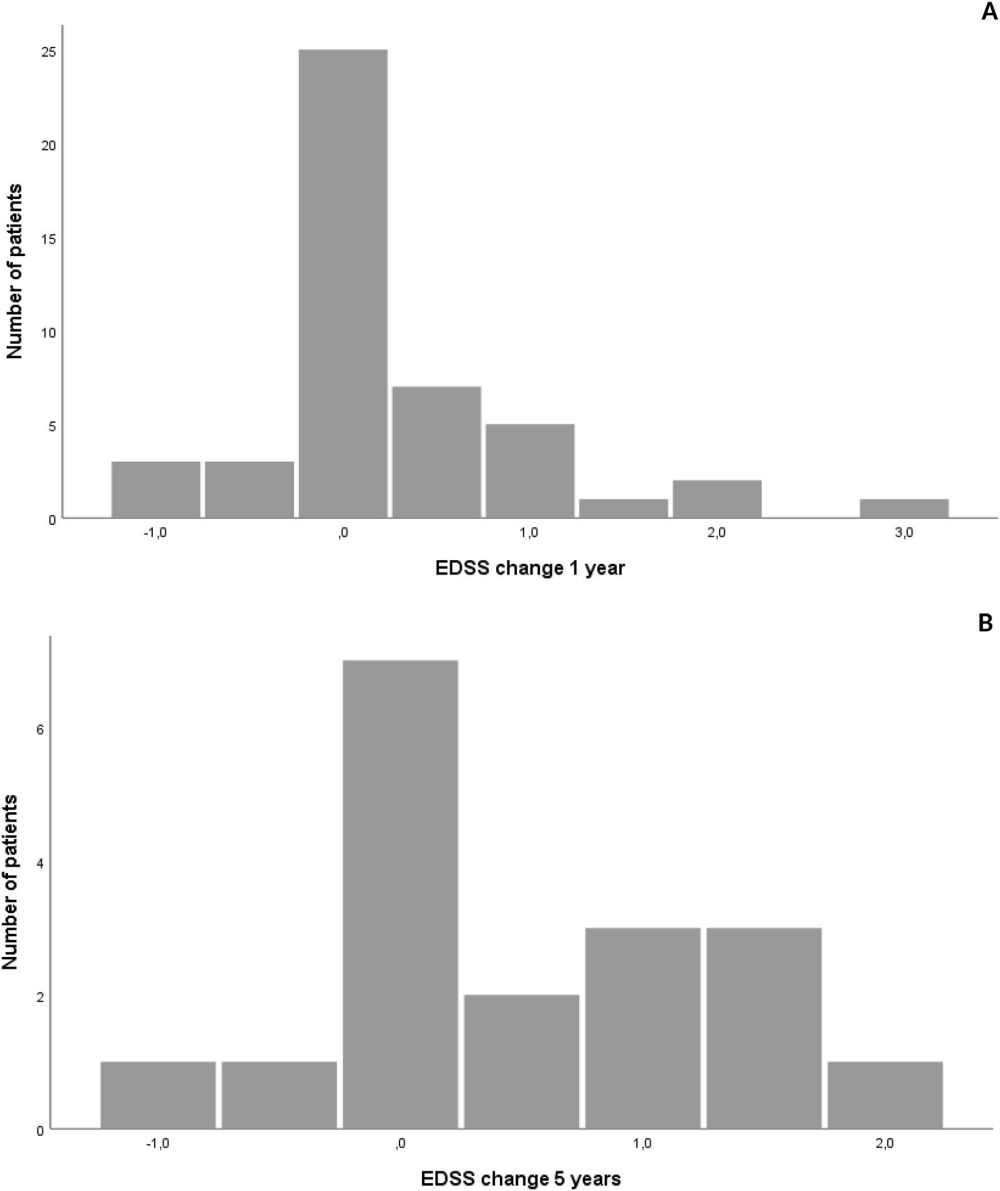
Change of EDSS score after NTZ discontinuation. Change of EDSS score within one (**A**, 47 patients) and 5 years (**B**, 18 patients) from time of NTZ discontinuation. Only patients with a NTZ treatment duration ≥ 2 years were considered.

In total, 105 of 235 patients at MUI discontinued NTZ until December 2020. The reasons for discontinuation are displayed in Table 2, where positive anti-JCV antibody status is the most common reason (55.2%) followed by secondary disease progression (17.1%). Most patients were switched to another DMT, most commonly to fingolimod (41.8%) followed by ocrelizumab (15.3%), and 12 (12.2%) patients did not receive any DMT after discontinuation of NTZ. For details, see Table 3. In patients who were on NTZ for at least 2 years, we analyzed the EDSS progression after treatment discontinuation. For this analysis, EDSS scores were available for 47 patients at 1 year and 18 patients at 5 years after NTZ discontinuation (Fig. 5). After 1 year, 16 of 47 patients (34.0%) experienced an EDSS worsening of at least 0.5 points, while after 5 years, this proportion was 9 out of 18 (50.0%).

**Table 2:** Reason for NTZ discontinuation.

Reason for discontinuation	N (%)
JCV positive	58 (55.2)
SPMS	18 (17.1)
Disease activity	8 (7.6)
NAbs	7 (6.7)
By request of patient	6 (5.7)
Comorbidity	6 (5.7)
Pregnancy	4 (3.8)
PML	3 (2.9)
Adverse event	3 (2.9)

105 patients discontinued NTZ. Those patients who stopped treatment due to pregnancy are planned to restart NTZ after delivery.

*JCV positive* positive for Anti-JC-Virus antibodies, *SPMS* secondary progressive multiple sclerosis, *NAbs* neutralizing antibodies, *PML* progressive multifocal leukoencephalopathy.

**Table 3:** First DMT after NTZ.

DMT after NTZ	N (%)
Fingolimod	41 (41.8)
Ocrelizumab	15 (15.3)
No DMT	12 (12.2)
Glatirameracetate	9 (9.2)
Dimethylfumarate	7 (7.1)
Rituximab	6 (6.1)
Siponimod	2 (2.0)
IVIG	2 (2.0)
IFN-beta	2 (2.0)
Mitoxantrone	1 (1.0)
Cyclophosphamide	1 (1.0)

For 98 patients data about further treatment strategy after discontinuation of NTZ were available.

*DMT* disease modifying treatment, *IVIG* intravenous immunoglobulins, *IFN* interferon.

For 59 patients, documentation of any relapses and MRI data were available. Of these, 32 (54.2%) experienced at least one relapse after NTZ discontinuation while 35 (59.3%) had MRI activity, 25 of whom also demonstrated contrast-enhancing lesions.

In the univariate analysis, significant differences between patients with and without disease reactivation were obtained for EDSS at treatment discontinuation (Mann–Whitney U test, p = 0.031, Z = − 2.156, r = − 0.28) and conversion to secondary progressive MS (SPMS) (chi-square test, p = 0.005, φ = − 0.37).

Finally, these 59 patients were selected for regression models for identifying risk factors for disease reactivation. The results are shown in Table 4.

**Table 4:** Regression models for potential risk factors for disease reactivation (A, binomial logistic regression) and time to first relapse (B, Cox regression) after NTZ discontinuation.

Variable	p values	OR	95% CI
**(A)**			
Gender (female)	0.216	3.63	0.47–28.03
Age	0.391	0.96	0.86–1.06
Disease duration	0.072	1.27	0.98–1.65
ARR before NTZ	0.226	1.78	0.70–4.58
Duration of NTZ treatment	0.685	0.94	0.68–1.29
Number of relapses before NTZ	0.844	1.03	0.74–1.44
Number of relapses on NTZ	0.749	0.70	0.08–6.09
ARR on NTZ	0.816	3.31	0.00–80,615
EID	0.218	0.32	0.54–1.95
SPMS	0.030	0.08	0.01–0.79
Number of DMTs before NTZ	0.222	2.11	0.64–6.99
**(B)**			
Gender (female)	0.098	3.15	0.81–12.24
Age	0.866	1.01	0.94–1.08
Disease duration	0.771	1.02	0.89–1.17
ARR before NTZ	0.014	1.46	1.08–1.96
Duration of NTZ treatment	0.566	1.08	0.84–1.38
Number of relapses before NTZ	0.139	0.83	0.65–1.06
Number of relapses on NTZ	0.626	1.44	0.33–6.17
ARR on NTZ	0.718	0.34	0.01–120.21
EID	0.928	0.92	0.14–6.13
SPMS	0.336	0.26	0.02–4.01
Number of DMTs before NTZ	0.135	2.17	0.79–5.98

A: For binomial linear regression model patients who discontinued NTZ after ≥ 2 years of treatment duration were divided in patients with and without occurring disease reactivation expressed by relapses and/or MRI activity after NTZ discontinuation. This model focused on risk factors whether there was a disease reactivation after stop of NTZ or not.

B: In addition, Cox regression model was performed in order to investigate potential risk factors for time to first relapse after NTZ withdrawal.

*ARR* annualized relapse rate, *EID* extended interval dosing before discontinuation, *SPMS* secondary progressive multiple sclerosis, *DMT* disease modifying treatment, *OR* odds ratio, *CI* confidence interval.

Both tables show significances (p-values) and odds ratio for each analysed potential risk factor.

The only significant result regarding whether disease reactivation occurs (Table 4A) was obtained for conversion to SPMS, which was shown to be protective (OR 0.08, 95% CI 0.01–0.79, p = 0.03) for disease reactivation after NTZ discontinuation while no other variables were found to be significant potential risk factors in this model.

Results of the Cox regression model for time to first relapse after NTZ are shown in Table 4B and reveal ARR before NTZ as the only significant risk factor (OR 1.46, 95% CI 1.08–1.96, p = 0.014) for early relapse after treatment discontinuation.

Regarding MRI data and EDSS, there were not enough data for performing similar multivariate calculations on the time variable.

In the univariate analysis for EDSS progression after NTZ discontinuation performed on 43 patients, only treatment duration (p = 0.038, Z = − 2.077, r = − 0.32) revealed a significant correlation.

## Discussion

With this retrospective cohort analysis, we aimed to describe the use of NTZ, its efficacy and the risk factors for disease reactivation after discontinuation in clinical practice outside of controlled studies. By including all available source data collected over decades, we were able to generate a very complete dataset with little missing data, allowing detailed analyses of clinical history over a long period of time. In contrast to most multicenter registries, this monocentric approach includes a large number of variables and reduces reporting bias while it also limits the number of patients and, consequently, the power of some analyses. Therefore, this study has some advantages regarding studying clinical practice and individualized treatment decisions.

We present a cohort of 235 NTZ-treated patients representing a typical MS cohort: predominantly female and aged approximately 33 years at NTZ start^23^. Disease history before the start of NTZ varies broadly among individual patients, showing a median of four relapses and 5 years of disease duration and a median EDSS of 2.0 at the time of NTZ start. One central objective of this study was to explore changes in clinical practice over time. There is a trend that NTZ as a highly effective DMT is now used earlier during the disease course, as indicated by fewer relapses and a lower EDSS at NTZ start as compared to the early treatment era. This is in line with current expert opinions which favor early highly effective treatments in active MS patients^24,25^, showing that this approach has been gradually adopted in clinical practice. Since the dependence of disease activity parameters from time of NTZ start was analyzed using a linear regression model, one has to take into account the limits of a classical linear regression that corrects only for fixed values and not for random effects.

Regarding treatment efficacy, our findings agree with the well-known results from controlled studies^2,3^ as well as the reports from other real-life cohorts^26^. For efficacy analysis, we included patients with a minimum treatment duration of 2 years. Since relapses during treatment with NTZ occur rarely, a longer observation period is favorable for data validity and less risk of overestimating ARR due to single relapses, especially around the time of treatment change. We found a strong relative reduction of ARR by 93%, while 64% of patients had a stable or improved EDSS score during the whole treatment period similar to other observational analyses^26,27^ that had comparable median treatment durations to our study. As a reference center for the whole region of Western Austria, we reported three cases of PML, two of which were referred from other hospitals, which explains the high proportion of PML in a relatively small cohort. As EID became an option for possible PML risk reduction^16,28,29^, 90% of a total of 40 patients who switched from standard to EID did not experience disease activity during the EID period. However, the median EID duration was short at less than 1 year in this subpopulation, which limits the validity of this analysis.

Finally, we focused on those patients who discontinued NTZ in whom positive anti-JCV antibody status after a treatment duration of 2 years or longer was the leading reason for NTZ discontinuation. Many patients were switched to fingolimod, probably because this has been the first option of an effective treatment besides injectables and NTZ since 2011. The switch from NTZ to fingolimod has been investigated in various studies^30,31^, most of which deemed this strategy relatively safe. Other highly effective drugs have been approved, especially B cell-depleting therapies such as rituximab, ocrelizumab and ofatumumab^15,32,33^, and have become another promising option for a switch from NTZ so that ocrelizumab was the second most used drug in our cohort after NTZ discontinuation. In the meantime, more data about the switch from NTZ to other recently approved and highly effective DMT are available, providing different promising exit strategies after withdrawal from NTZ^14^.

Different studies have tried to identify risk factors for disease reactivation after stopping NTZ, with some inconsistent results^17,34–38^. In most of these studies, risk factors for disease reactivation were longer washout periods of NTZ, higher relapse rate before NTZ and, inconsistently, younger age, higher baseline EDSS before NTZ and higher disease activity on NTZ measured by relapses and EDSS progression. Butzkueven et al.^34^ also identified shorter treatment duration as an additional risk factor. While Lo Re et al.^36^ assumed a lower risk of disease reactivation on fingolimod compared to interferon-beta, glatirameracetate and teriflunomide, Capobianco et al.^39^ did not report an impact of the DMT chosen after NTZ.

In our univariate analysis, conversion to SPMS and higher EDSS at NTZ discontinuation were associated with lower risk of disease reactivation and to some extent, shorter treatment duration as well. However, in the multivariate models performed on patients who were treated for at least 2 years, none of the above variables were independent risk factors except conversion to SPMS. Furthermore, annual relapse rate before NTZ was associated with earlier disease reactivation in those patients who experienced at least one relapse after NTZ discontinuation.

In this context, we have to take into account the statistical limitations of the chosen regression models which arose due to the amount and quality of available data, especially due to the retrospective study design that limits the use of the variable time, which has not always been exactly documented (e.g., intervals of MRIs performed and intervals between single visits varied among the patients). Therefore, we first used a binary logistic regression to answer the question of whether (and not primarily when) disease activation expressed by relapses or MRI activity occurred. In a second step, we applied the Cox regression considering those patients where the variable time was exactly documented and choosing relapse as the outcome variable, being aware that with perfectly, consistently and perhaps prospectively collected data, the use of the Cox regression alone would have been favorable.

Besides statistical concerns, there may be several reasons for the aforementioned inconsistent results among different studies and for discrepancies between those and our study. Most of these studies identified the treatment gap between NTZ and subsequent DMT as a risk factor. In this regard, our analysis may be limited by the low number of patients, and treatment gaps were usually less than 3 months in our cohort while other studies had a considerable number of patients with longer intervals between NTZ and following DMT, which allowed them to demonstrate significant influence of this variable. Moreover, the selection of patients for multivariate analyses differed among studies. For example, Butzkueven et al.^34^ only included patients who switched to an oral therapy after NTZ. Mustonen et al.^35^ calculated their model case by case according to the subsequent DMT. Salhofer-Polanyi et al.^38^ analyzed a cohort of patients of whom 50% had a treatment duration of less than 2 years. Vidal-Jordana et al.^37^ chose significant clinical worsening by EDSS progression after stopping NTZ for their multivariate model, which cannot be counted as immediate reactivation. In Lo Re et al.’s^36^ study, only in univariate analyses were the described variables significantly associated with disease activity after NTZ. However, conversion to SPMS was not included as a variable in all these studies. Prosperini et al.^17^ included further studies in their review, finding poor evidence of any risk factor for disease reactivation, which is in line with our results. Altogether, there is substantial variation regarding study designs and choice of outcome variables across the different studies, which makes it difficult to identify reliable risk factors.

According to our data, patients who convert to progressive MS during treatment could be more safely withdrawn from NTZ independent from further treatment strategy, while for all patients who are stable on NTZ without signs of disease progression, there are no clear risk factors for post-NTZ disease activity. Patients with a high annual relapse rate before NTZ seem to be at risk for earlier disease reactivation after ceasing NTZ treatment, so one should consider short time intervals to the start of alternative treatment, especially in those patients.

## Methods

### Patients and data acquisition

At MUI, an electronic MS database was established in 2004 including all patients treated at the MS outpatient clinic. This database includes data such as demographics, details of MS course, diagnostic investigations, treatment history, EDSS and onset of secondary progression^40^.

This database was used for preselection of patients, generating a data acquisition file including all patients who were ever treated with NTZ. In order to gather as much and as exact of data as possible, in a second step, we searched all source data (i.e., the hospital’s medical records). In this way, we created a final data file for further analyses.

For pretreatment analysis, all patients were included who had at least one visit after NTZ treatment start and where treatment history such as date of diagnosis, number of relapses, DMTs and date of NTZ start were sufficiently recorded. On-treatment analysis was performed on patients who had a treatment duration of at least 2 years and were continuously followed up with every 3–6 months during treatment. The same criteria were used for discontinuation analysis. Disease reactivation after NTZ discontinuation was defined by relapses or MRI activity (i.e., new T2 or contrast-enhancing lesions).

### Ethical approval

The study was approved by the ethical committee of MUI, Austria (approval number 1033/2021). Permission to take informed consent was formally waived by the approving ethical committee. In this retrospective study, all analyses were performed with pseudonymized data, which did not require written informed consent based on applicable ethical practice and the statement of the ethical committee which approved the study. All methods were performed in accordance with the relevant guidelines and regulations.

### Statistics

Statistical analysis was performed using SPSS 26.0 (IBM, Armonk, NY, USA). Shapiro–Wilk and Kolmogorov–Smirnov normality tests were used simultaneously to analyze the distribution of data. Descriptive data are displayed as median and IQR or range as indicated or mean and standard deviation as appropriate. Depending on type and distribution of data, Mann–Whitney U tests, t tests or chi-square tests were used for group comparison. Correlations were performed using Pearson correlation coefficients and Z-scores.

In the pretreatment analysis, we aimed to investigate the clinical practice regarding the timepoint when NTZ was started during the disease course and whether this practice changed from approval of the drug to the present. Therefore, we built a linear regression model to analyze whether the covariates EDSS at treatment start, number of relapses before NTZ start and disease duration at time of NTZ start, which represent disease activity parameters (dependent variables), changed dependent on time of treatment start (independent variable).

Regarding the association of different variables with disease reactivation, in the discontinuation analysis, we started with univariate analysis of all clinical and demographical variables between patients with and without disease reactivation using Mann–Whitney U or Chi-square tests as appropriate. EID duration, EDSS at NTZ start and discontinuation as well as the interval between NTZ and the subsequent DMT were not significantly associated and were finally excluded from the subsequent regression models because they were only available for some patients and thus influenced the quality of the models (AIC value). A detailed analysis for single DMTs or moderate versus highly active DMTs was not expedient due to small subgroups and therefore missing statistical power.

Thereafter, two steps of regression models were used for multivariate analysis in order to address two different central questions. First, we aimed to analyze possible variables which could predict whether there would be a disease reactivation after NTZ discontinuation. Therefore, a binomial logistic regression was performed which employed all the variables listed in Table 4A as potential influencing factors and disease reactivation (i.e., relapses and/or MRI activity) as the dichotomic outcome.

Second, a Cox proportional hazard regression model for identifying risk factors for early relapses after treatment cessation was calculated. This model should address the question about the time to first relapse after treatment cessation and again employ the variables listed in Table 4B as potential influencing factors.

Two-tailed p-values < 0.05 were considered statistically significant.

## Data Availability

All data are available upon request from the corresponding author.
